# A Mouse Model of Human Primitive Neuroectodermal Tumors Resulting from Microenvironmentally-Driven Malignant Transformation of Orthotopically Transplanted Radial Glial Cells

**DOI:** 10.1371/journal.pone.0121707

**Published:** 2015-03-31

**Authors:** Sergey Malchenko, Simone Treiger Sredni, Hitoshi Hashimoto, Atsushi Kasai, Kazuki Nagayasu, Jianping Xie, Naira V. Margaryan, Kaoru Seiriki, Rishi R. Lulla, Richard E. B. Seftor, Lauren M. Pachman, Herbert Y. Meltzer, Mary J. C. Hendrix, Marcelo B. Soares

**Affiliations:** 1 Cancer Biology and Epigenomics Program, Stanley Manne Children’s Research Institute, Ann & Robert H. Lurie Children’s Hospital of Chicago, Chicago, Illinois, United States of America; 2 Department of Pediatrics, Feinberg School of Medicine, Northwestern University, Chicago, Illinois, United States of America; 3 Robert H. Lurie Comprehensive Cancer Center, Feinberg School of Medicine, Northwestern University, Chicago, Illinois, United States of America; 4 Division of Pediatric Neurosurgery, Department of Surgery, Ann & Robert H. Lurie Children’s Hospital of Chicago and Feinberg School of Medicine, Northwestern University, Chicago, Illinois, United States of America; 5 Laboratory of Molecular Neuropharmacology, Graduate School of Pharmaceutical Sciences, Osaka University, 1–6 Yamadaoka, Suita, Osaka, Japan; 6 iPS Cell-based Research Project on Brain Neuropharmacology and Toxicology, Graduate School of Pharmaceutical Sciences, Osaka University, 1–6 Yamadaoka, Suita, Osaka, Japan; 7 Molecular Research Center for Children’s Mental Development, United Graduate School of Child Development, Osaka University, Kanazawa University, Hamamatsu University School of Medicine, Chiba University and University of Fukui, Osaka University, 1–6 Yamadaoka, Suita, Osaka, Japan; 8 Interdisciplinary Program for Biomedical Sciences, Institute for Academic Initiatives, Osaka University, 1–1 Yamadaoka, Suita, Osaka, Japan; 9 Division of Hematology/Oncology, Department of Pediatrics, Feinberg School of Medicine, Northwestern University, Chicago, Illinois, United States of America; 10 Cure-Juvenile Myositis Program of Excellence, Division of Pediatric Rheumatology, Ann & Robert H. Lurie Children’s Hospital of Chicago and Feinberg School of Medicine, Northwestern University, Chicago, Illinois, United States of America; 11 Psychiatry & Behavioral Sciences at the Feinberg School of Medicine, Northwestern University, Chicago, Illinois, United States of America; The University of Tokyo, JAPAN

## Abstract

There is growing evidence and a consensus in the field that most pediatric brain tumors originate from stem cells, of which radial glial cells constitute a subtype. Here we show that orthotopic transplantation of human radial glial (RG) cells to the subventricular zone of the 3rd ventricle - but not to other transplantation sites - of the brain in immunocompromised NOD-SCID mice, gives rise to tumors that have the hallmarks of CNS primitive neuroectodermal tumors (PNETs). The resulting mouse model strikingly recapitulates the phenotype of PNETs. Importantly, the observed tumorigenic transformation was accompanied by aspects of an epithelial to mesenchymal transition (EMT)-like process. It is also noteworthy that the tumors are highly invasive, and that they effectively recruit mouse endothelial cells for angiogenesis. These results are significant for several reasons. First, they show that malignant transformation of radial glial cells can occur in the absence of specific mutations or inherited genomic alterations. Second, they demonstrate that the same radial glial cells may either give rise to brain tumors or differentiate normally depending upon the microenvironment of the specific region of the brain to which the cells are transplanted. In addition to providing a prospect for drug screening and development of new therapeutic strategies, the resulting mouse model of PNETs offers an unprecedented opportunity to identify the cancer driving molecular alterations and the microenvironmental factors that are responsible for committing otherwise normal radial glial cells to a malignant phenotype.

## Introduction

RG cells, primitive neuroectoderm progeny, are thought to be the progenitor cells for adult neural stem cells (NSC), neurons, basal progenitors, astrocytes and oligodendrocytes, in addition to being responsible for the majority of neurogenesis in the developing brain [1]. Pediatric brain tumors—such as ependymomas—have also been shown to derive from RG cells [2–5]. Recently we reported a new approach that allowed us to derive large amounts of RG cells from human embryonic stem cell (hESC), and from human induced pluripotent stem cell (hiPSC) lines. We demonstrated that RG cells orthotopically transplanted to the motor cortex of 8-week old immunocompromised NOD-SCID mice can differentiate into functionally active, mature-appearing pyramidal and serotonergic neurons [6].

In the present study we orthotopically transplanted RG cells to different brain regions of NOD-SCID mice, including the subventricular zone (SVZ) of the 3rd ventricle, at a site that is in close proximity to the lateral ventricles—one of the preferential sites of brain tumor formation [3, 7, 8]. It is noteworthy that the SVZ of the 3rd ventricle has recently been identified as a potentially new site of pediatric glioma formation [8]. We used a panel of RG cell lines derived from hESCs, and from iPSCs that were generated using mononucleocytes obtained from patients with aggressive medulloblastoma, low-grade glioma, germinoma, from a psychiatric patient (with no history of cancer development), as well as from a healthy child. The objectives of this study were two-fold: to monitor the differentiation of RG cells in their “natural” microenvironment, and to investigate whether radial glial cells derived from patients with brain tumors harbor genetic/genomic alterations that might commit them to tumorigenesis [9, 10].

## Materials and Methods

### Ethics statement

Written informed consents were obtained prior to blood sample collection for this study. Establishment of all hiPSC lines was approved by the Stanley Manne Children's Research Institute Institutional Review Board. All animal-related procedures were approved by the Institutional Animal Care and Use Committee accredited by the Association for Assessment and Accreditation of Laboratory Animal Care and conformed to the standards of the National Institutes of Health (IACUC protocol #: 2011–09). Standard consenting procedures were applied in this study. For all patients under 18 years of age, written parental consent was obtained. For all patients over 12 years of age, an additional written assent was obtained. No oral consents or assents were obtained. The consent process was documented in our electronic medical record system. This study was conducted under the Institutional Review Board approved protocol numbers: 2001–11715, 2012–14877, STU00072711).

### Derivation of HiPSC and Radial glial (RG) cell lines

Five hiPSC lines: LC25, LC26, LC30, LC35TR and LCAS were established at the Stanley Manne Children’s Research Institute and used for this study. The cell lines were generated using peripheral blood mononuclear cells obtained from patients with germinoma (male, 12 y/o), aggressive medulloblastoma (female, 5 y/o), low grade glioma (female, 7 y/o), a psychiatric patient with no history of cancer development (male, 34 y/o), and from a healthy child (male, 9 y/o) respectively, by over-expressing Oct3/4, Sox2, KLF4 and cMyc using CytoTune-iPS Sendai Reprogramming Kit according to the manufacturer’s protocol (Gibco). Derivation of RG cell lines LC25-R, LC26-R, LC30-R, LC35TR-R and LCAS-R (previously called, *Rosette neural stem cell lines*), flow cytometry analysis of the cell lines, and immunohistochemistry of the brain tissue slides (50um) were performed as previously described [6]; for the latter, a 1:50 dilution of antibodies against Ki67 (AbCam) was utilized. We also used radial glial cell line CM14R, which was derived from human embryonic stem cells as previously described [6]. GFP labeling was performed using the vector system described in [11].

### RG cell culturing in hypoxic conditions

The only difference in the cell culturing conditions was the concentration of O_2_: the RG cells were cultured for 72 hours in hypoxic conditions (5% O_2_), along with normoxic conditions (20% O_2_).

### Orthotopic transplantation of RG cells to brain regions of NOD-SCID mice

Transplantations of the RG cells to the selected areas of the brain of NOD-SCID mice were performed as previously described [6]. Briefly, transplantations of RG-GFP cells to target SVZ of 4th ventricle in cerebellum, motor cortex or SVZ of 3rd ventricle were performed as follows: a 1.0mm burr hole was made approximately -7.0mm dorsal caudal from the bregma, 2.0mm dorsal caudal, 0.8mm right or left lateral from the bregma, and 0.3mm dorsal caudal from the bregma. A 26 gauge needle attached to a 25 μl Hamilton syringe was inserted into the depth of 3.0mm, 2.0mm, and 4.0mm correspondingly from the skull surface using stereotactic guidance. Five microliters containing 200,000 (-7.0mm dorsal caudal from the bregma), 3.5x10^5^, 2.0x10^5^, or 5x10^4^ (2.0mm dorsal caudal, 0.8mm right or left lateral from the bregma), 2.0x10^5^, 1.0x10^5^, or 5x10^3^ (0.3mm dorsal caudal from the bregma) of the RG-GFP cells were inoculated into the brain over a period of 10 minutes.

The mice were sacrificed and brains harvested at 4–12 weeks post-inoculation. All the harvested brains were perfused with 4% paraformaldehyde as previously described [6].

### Immunohistochemistry (paraffin slides 5 μm)

Half of the tumors (along with the corresponding RG-GFP cells) were fixed in 10% formalin for 48 hours and embedded in paraffin. The samples were stained by hematoxylin and eosin (HE) for histologic examination. The immunohistochemical panel comprised antibodies against OTX2 (AB9566, Rabbit Polyclonal, 1:750, Millipore), BLBP (ABN14, Rabbit Polyclonal, 1:400, Millipore), N-Cadherin (D-4, sc-8424, Mouse Monoclonal, 1:30, Santa Cruz), Ki-67 (RM-9106, Rabbit Monoclonal, 1:200, Thermo scientific), B-Catenin (E247, MA5-14461, Rabbit Monoclonal, 1:50, Thermo Scientific), TJP1 (LS-C186010, Rabbit Polyclonal, 1:250, LSBio), and TJP3 (LS-B2896, Rabbit Polyclonal, 1:50, LSBio).

### Western blotting

Whole cell lysates were prepared in RIPA buffer (100 mM Tris/HCl, pH 7.5, 150 mM NaCl, 1% deoxycholate, 1% Triton X-100, 0.1% SDS) plus protease inhibitors (Complete Mini, Roche Diagnostics, Indianapolis, IN) with sonication using three, three second pulses. After centrifugation at 13K rpm for 30 minutes at 4°C, the protein concentration of the supernatant was determined using a bicinchoninic acid assay (Thermo Scientific, Rockford, IL) and 40μg of total protein loaded per well of a Novex 4–12% NuPAGE Bis-Tris gel (Life Technologies, Carlsbad, CA). After electrophoresis, the proteins were transblotted onto a PVDF membrane (Bio-Rad Laboratories, Hercules, CA) and TJP1 protein detected using a mouse monoclonal antibody (NBP1-51708, 1:500, Novus Biologicals, Littleton, CO), donkey anti-rabbit antibody linked to horseradish peroxidase (NA934V, GE Healthcare, Little Chalfont Buckinghamshire, UK) and ECL substrate (Western Bright ECL, Advansta, Menlo Park, CA). The blot was then stripped and probed for β-Actin protein using a mouse monoclonal antibody (MAB1501, Millipore, Temecula, CA), sheep anti-mouse antibody linked to horseradish peroxidase (NA931V, GE Healthcare) and ECL substrate. Densitometry of the protein bands were measured using a ChemiDoc XRS imager with Quantity One software package (Bio-Rad Laboratories) and the differences in TJP1 protein calculated after correcting for actual protein loaded per lane using the β-Actin protein control.

### Whole brain imaging

Whole brain imaging was performed using a microscope (Eclipse FN1, Nikon, Tokyo, Japan) equipped with a spinning disk confocal unit (CSU-W1, Yokogawa Electric Corp., Tokyo, Japan) and an electron-multiplying charge-coupled device camera (iXon 3, Andor Technology, Tokyo, Japan). Three-dimensional reconstruction from coronal section images of the brain every 50 μm were carried out using iQ software (Andor) and Fiji/ImageJ software (1.49a, NIH; Bethesda, NJ).

## Results and Discussion

**Fig 1:**
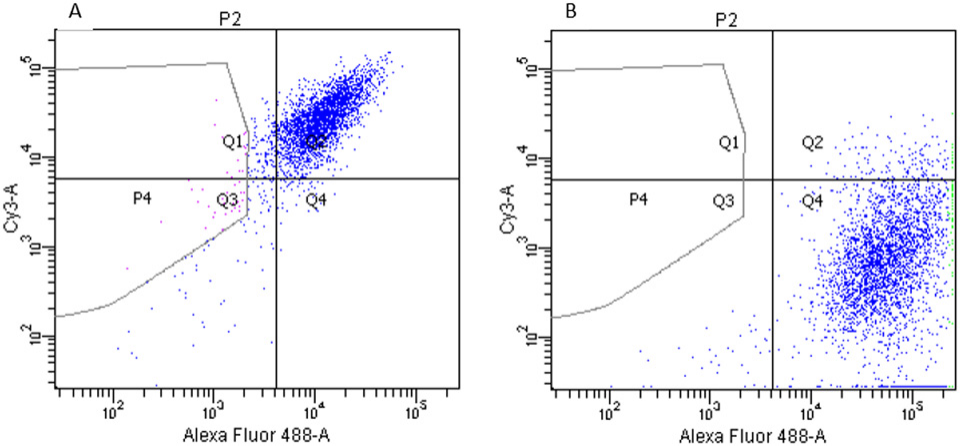
Flow cytometry analysis of the wild type RG cells before labeling them with GFP. A- Double staining with Sox2 (488-A) and Nestin (Cy3-A): Q1-Nestin (4.1%), Q2-Nestin: Sox2 (90.2%), Q4-Sox2 (1.9%), Q3 (negative control). B- Double staining with BLBP (488-A) and Tubb3 (Cy3-A): Q1-Tubb3 (0.0%), Q2-Tubb3: BLBP (4.5%), Q4-BLBP (94.4%), Q3 (negative control).

**Fig 2:**
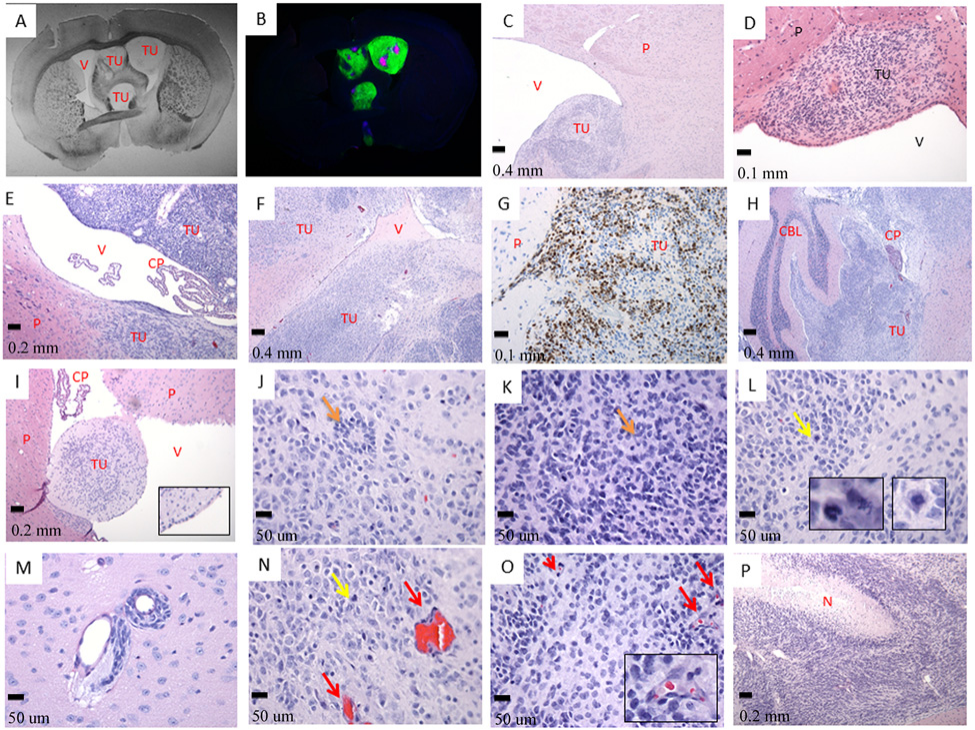
The tumor cells invade the ventricular system. A- Septo-Striatal section, phase contrast (tissue slides, 1X); B- The same Septo-Striatal section: Ki-67 (red) overlay with GFP (green) and DAPI (blue) (LC25-R—8 weeks post-injection) (tissue slides, 1X); C- (LC26-R—12 weeks post-injection), D- (LC35TR-R—12 weeks post-injection), and E- (LCAS-R—12 weeks post-injection)—tumor growing within the parenchyma of the subventricular zone protruding to the ventricle (HE- 5X, 20X and 10X respectively); F- tumor growing within the parenchyma and invading lateral ventricle (LC26-R—12 weeks post-injection) (HE- 5X); G- tumor growing within the parenchyma, Ki-67 staining (LC26-R—12 weeks post-injection) (HE- 20X); H- tumor growing within the ventricular system including forth ventricle and subarachnoid space permeating the cerebellum (LC26-R—12 weeks post-injection) (HE- 5X); I- Tumor growing inside the parenchyma and protruding into the ventricular space. (CM14R - 8 weeks post-injection) (HE- 10X, inserts- 4X digital); J- and K- poorly differentiated neuroblastic tumor with rosette formation (LC26-R—12 weeks post-injection) (HE- 40X), rosettes—orange arrows; L- and N- frequent atypical mitoses—yellow arrows and inserts (LC26-R—12 weeks post-injection) (HE- 40X, inserts- 6X digital); M- tumor perivascular invasion (LCAS-R—12 weeks post-injection) (HE— 40X); N- and O—prominent angiogenesis—red arrows and insert (LC26-R—12 weeks post-injection) (HE- 40X, insert- 5X digital); P- geographic necrosis with pseudo-palisades (LCAS-R—12 weeks post-injection) (HE— 10X).TU—tumor; P—parenchyma; V—ventricle; CP—choroid plexus; CBL—cerebellum; N—necrosis.

All RG cell lines exhibit normal karyotypes and express Sox2-, Nestin-, and BLBP (Fig 1). Surprisingly, all RG cell lines, including those derived from the healthy child, the psychiatric patient, and the hESC line, gave rise to tumor masses (Fig 2A and 2B) [12].

**Fig 3:**
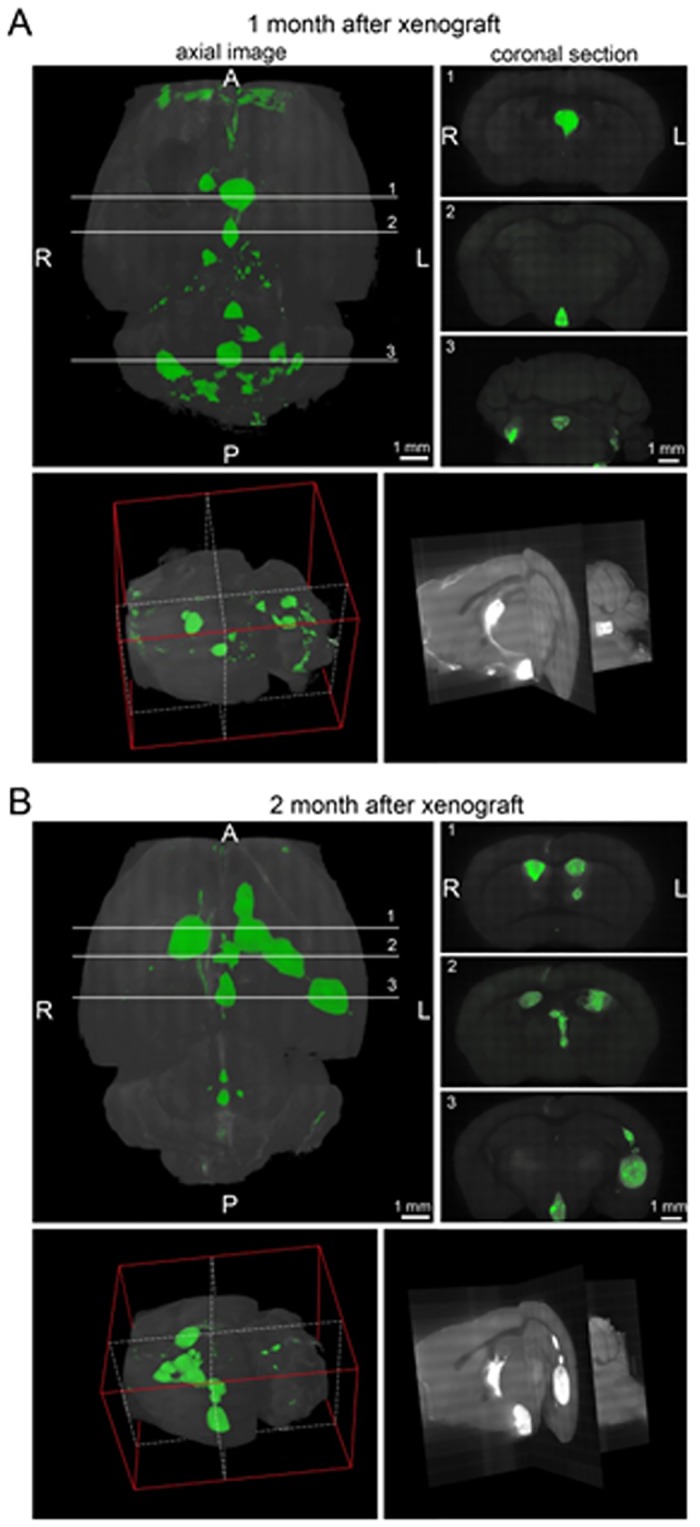
3D imaging of the mice brain using a confocal microscope at one month A- or two months B- post—injection (LCAS-R). Top left panels reconstitute images of the brains placed horizontally, top right panels reconstitute coronal sections at the indicated lines 1–3. Bottom left panels reconstitute 3D image of the brains oriented as shown by the red plot lines. The section images at the white dotted lines shown at the bottom right panels. A—anterior; P—posterior; R-right; L—left.

Histopathological analyses revealed hallmarks of tumors of neuroectodermal origin, including CNS primitive neuroectodermal tumors (PNETs). Tumors started growing within the site of transplantation at the subventricular zone, infiltrating the adjacent parenchyma (Fig 2C–2E). Subsequently, tumors invaded lateral ventricles (Fig 2I and 2F), ultimately occupying the ventricular system (Fig 3A and 3B, S1 File) with extension to the 4th ventricle and subarachnoid space, permeating the cerebellum (Fig 2H). All tumors originated from the RG cells have a similar histopathological phenotype characterized by densely arranged primitive cells with round to oval hyperchromatic nuclei, and scarce cytoplasm growing within a delicate neurofibrillary background. A widespread presence of neuroblastic (Homer Wright) rosettes with halo-like clusters of cells surrounding central neuropil-rich areas (Fig 2J and 2K) was also observed, suggesting the neuroectodermal nature of these tumors. All tumors presented a high proliferative index, as assessed by Ki-67 staining (Fig 2G).

In addition to a high nuclear-cytoplasmic ratio, intense proliferative activity, infiltrative growth, and invasion of the ventricular system, other aspects of malignancy were observed in these tumors. These include perivascular invasion (Fig 2M), presence of atypical mitoses [13, 14] (Fig 2L and 2N), widespread tumor angiogenesis reflected by vascular endothelial proliferation (Fig 2N and 2O), and necrotic zones surrounded by tumor cells arranged in pseudo-palisades (Fig 2P) [15].

**Fig 4:**
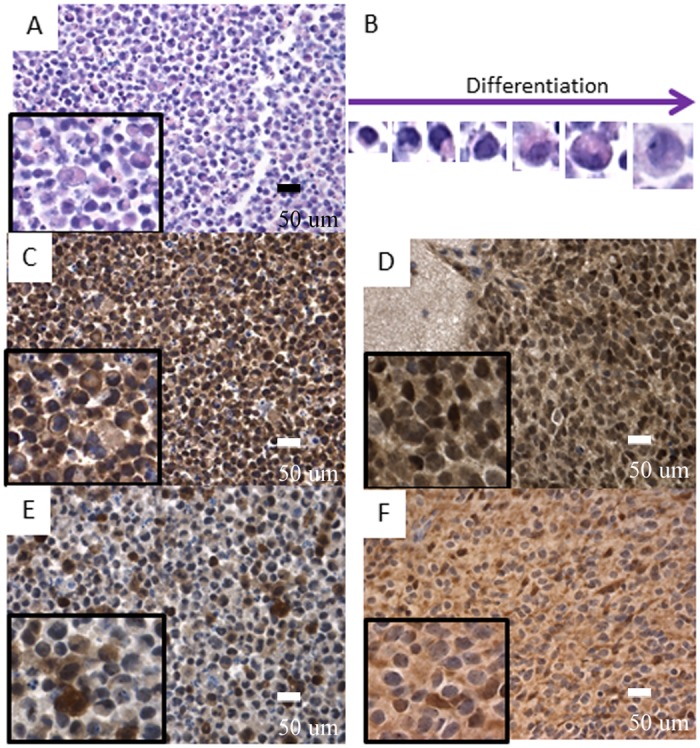
Polymorphic population of RG cells. A- and B- wild type RG cell line shows polymorphic population of cells with neuroectodermal differentiation (HE— 40X, inserts digital magnification 4X and 10X); C- and D- OTX2 expressed in early stages of differentiation among wild type RG cells (C— 40X, inserts digital magnification 4X) and in the majority of tumor cells (LCAS-R—12 weeks post-injection) (D— 40X, inserts digital magnification 4X); E- and F- BLBP expressed in neuronal differentiated cells within wild type RG cells (E— 40X, inserts digital magnification 4X) with loss of expression in most of the tumor cells (LCAS-R—12 weeks post-injection) (F— 40X, inserts digital magnification 4X).

In contrast to the histological appearance of the tumors that developed following orthotopic transplantation of RG cells, the analysis of each RG cell line before transplantation to NOD-SCID mice revealed polymorphic population of cells without evidences of malignancy. Although the majority of the cell population was represented by small cells with round nuclei and scant cytoplasm, a number of cells showed neuronal features with eosinophilic or amphophilic cytoplasm, large eccentric nuclei with vesicular chromatin, and single prominent nucleoli. The remainder of the cells was categorized into the spectrum of differentiating cells with variable amounts of cytoplasm and progressively enlarged nuclei (Fig 4A and 4B).

In order to characterize the neoplastic transformation of the RG cells more specifically, a panel of antibodies was tested on the RG cell lines and on their derived tumors, including an antibody directed against transcription factor OTX2, which may be considered as a PNET oncogene [16].

The results document that the RG cell lines express OTX2 at early stages of neuroectodemal differentiation, and that expression is lost as they transition towards neuronal differentiating cells (Fig 4C). In a similar manner, but inversely correlated, BLBP is expressed in the cytoplasm of neuronal differentiating cells (Fig 4E). The majority of tumor cells retain OTX2 expression homogeneously and diffusely (Fig 4D), while lose BLBP expression (Fig 4F), which is indicative of the neuroectodermal nature of these tumors.

**Fig 5:**
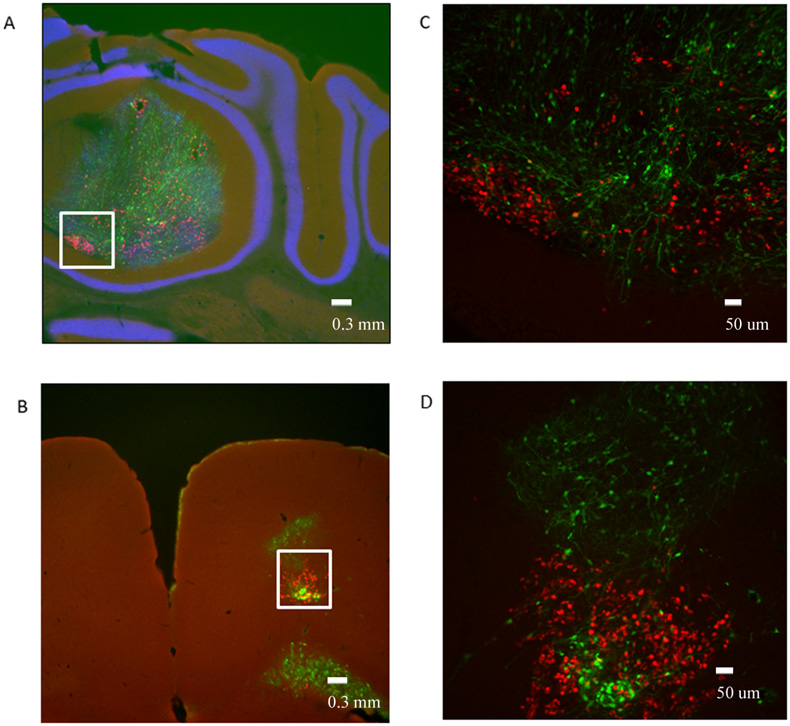
Orthotopic transplantation of the wild type RG cells to different brain areas of the mice. A- Cerebellum: Ki-67 (red) overlay with GFP (green) and DAPI (blue), white rectangle identifies area magnified in Fig 5C (LCAS-R—6 weeks post-injection) (tissue slides, 2.5X); B- Motor Cortex: Ki-67 (red) overlay with GFP (green) and DAPI (blue), white rectangle identifies area magnified in Fig 5D (LCAS-R—6 weeks post-injection) (tissue slides, 2.5X), C, D- Cerebellum an Motor Cortex: Ki-67 (red) overlay with GFP (green) (LCAS-R—6 weeks post-injection) (tissue slides, 10X).

**Fig 6:**
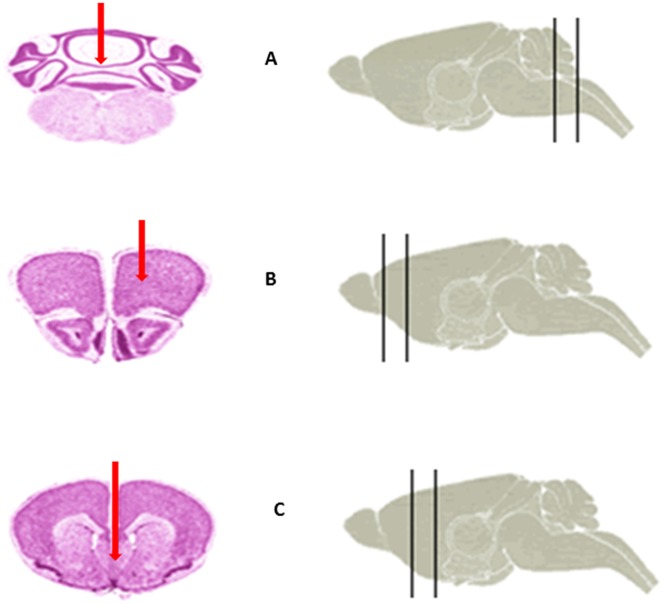
Illustration of the orthotopic transplantation sites (red arrows). A- Cerebellum. B- Motor cortex. C- SVZ of 3^rd^ ventricle. (The images were taken from Harvard Medical School High Resolution Mouse brain Atlas).

To investigate whether proximity to the ventricular system is the determining factor for the documented tumorigenic transition of RG cells, we orthotopically transplanted the control cells derived from the healthy child (LCAS-R) to the cerebellum, targeting the subventricular zone of the 4th ventricle. While displaying a high proliferative index, as assessed by Ki-67 staining, the transplanted cells predominantly remained at the transplantation sites, showing no evidence of malignant transformation or invasion into the ventricular system (Fig 5A and 5C). In parallel, the same control cells were transplanted to the motor cortex (Fig 5B and 5D), where they exhibited normal behavior—including proliferation, migration and differentiation—similar to that previously reported for another radial glial cell line [6]. Once again, there was no evidence of invasion of the ventricular system (Figs 5 and 6, Table 1).

**Table 1:** Summary of the coordinates for orthotopic transplantation sites.

	Cerebellum	Motor cortex	SVZ of 3rd ventricle
**Coordinates dorsal caudal from the bregma**	-7.0mm dorsal caudal from the bregma	2.0mm dorsal caudal from the bregma	0.3mm dorsal caudal from the bragma
**Coordinates lateral from the bregma**	0.0mm lateral from the bregma	0.8mm right or left lateral from the bregma	0.0mm lateral from the bregma
**Depth of the cells inoculation**	3.0mm	2.0mm	4.0mm
**Appearance of the tumors**	NO	NO	YES

**Fig 7:**
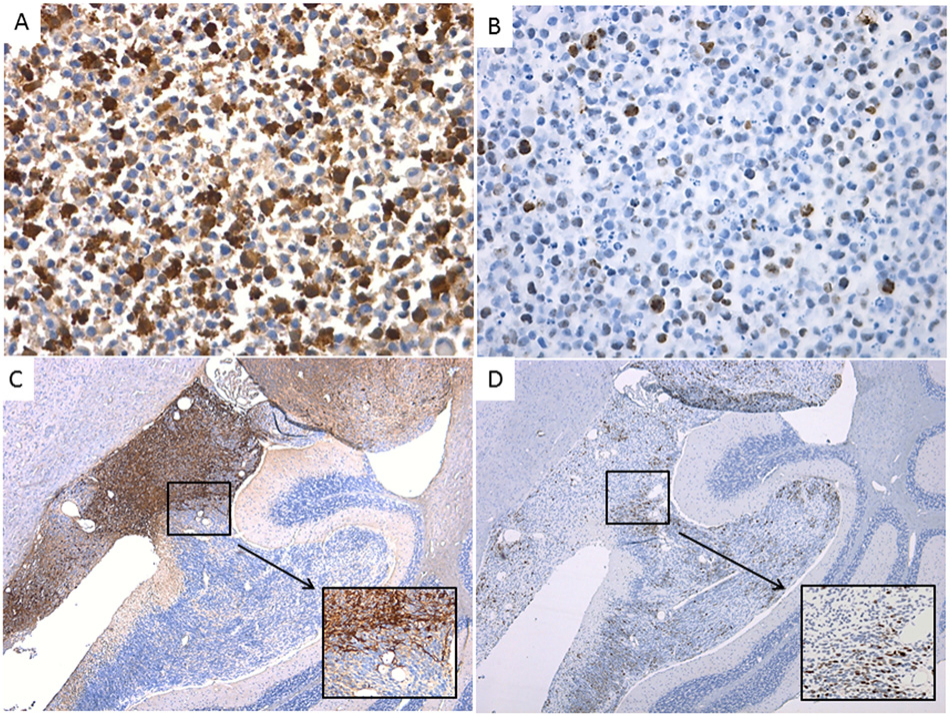
GFP and Ki-67 expression do not overlap in the majority of the cells in cell lines or in the tumors derived from them. A- LC25-R-p9, GFP (1:750), 40X; B- LC25-R-p9, Ki-67(1:200), 40X; C- LCAS-R, GFP (1:750), 5X (insert 40x digital); D- LCAS-R, Ki-67 (1:200), 5X (insert 40x digital).

It is noteworthy that the majority of the transplanted cells, at these two sites, did not show co-expression of GFP and Ki-67 (Fig 5), which we interpret to be due to the fact that the fast dividing Ki-67 positive cells are likely not have enough GFP in the cytoplasm to be detected by IHC. The same trend was observed both in the RG cell lines and in the tumors (Fig 7). As documented in Fig 3A and in the supplemental video, one month after transplantation the GFP positive tumor cells had already colonized the entire ventricular system. Hence, it seems plausible to hypothesize that the fast dividing Ki-67 positive cells might have been the first to invade the ventricular system, and that as the cells started to differentiate and consequently to slow down their division rates, GFP could accumulate in the cytoplasm and thus become detectable.

**Fig 8:**
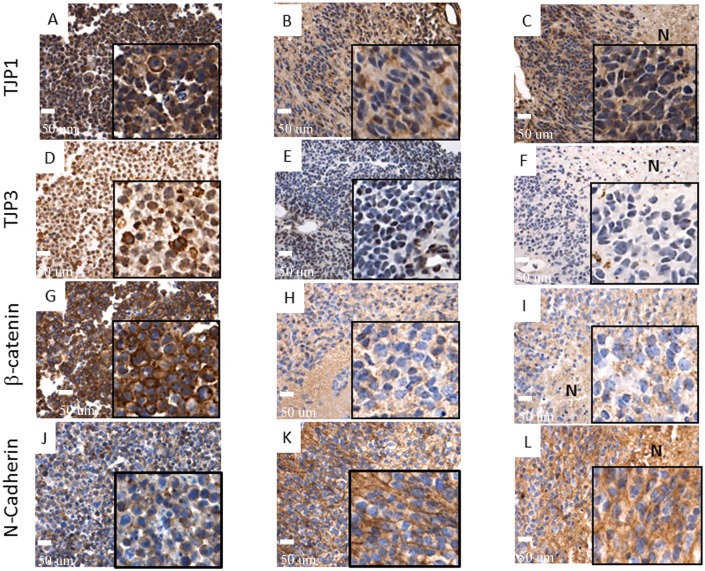
EMT markers show loss of expression by immunohistochemistry in tumors when compared to their corresponding wild type RG cell lines. TJP1 A- Diffuse membrane expression in wild type RG cells, B- Multifocal loss of expression in tumors, C- Loss of expression around necrotic areas (LCAS-R—12 weeks post-injection) (40X, inserts digital magnification 4X); TJP3 D- 2/3 of cells present cytoplasmic expression in wild type RG cells; E- Extensive areas of loss of expression in tumors (LCAS-R—12 weeks post-injection), F- Loss of expression in areas surrounding necrosis (LC26-R—12 weeks post-injection) (40X, inserts digital magnification 4X); β-catenin G- Diffuse membrane expression in wild type RG cells, H- Almost complete loss of expression in corresponding tumors (LCAS-R—12 weeks post-injection), I- Loss of expression in areas surrounding necrosis (LC26-R—12 weeks post-injection) (40X, inserts digital magnification 4X); N-cadherin, J- Low membrane expression in wild type RG cells, K- Extensive membrane expression in tumor cells (LCAS-R—12 weeks post-injection) (40X, inserts digital magnification 4X), L- Extensive membrane expression in the areas surrounding necrosis (LC26-R—12 weeks post-injection) (40X, inserts digital magnification 4X). N—Necrosis.

**Fig 9:**
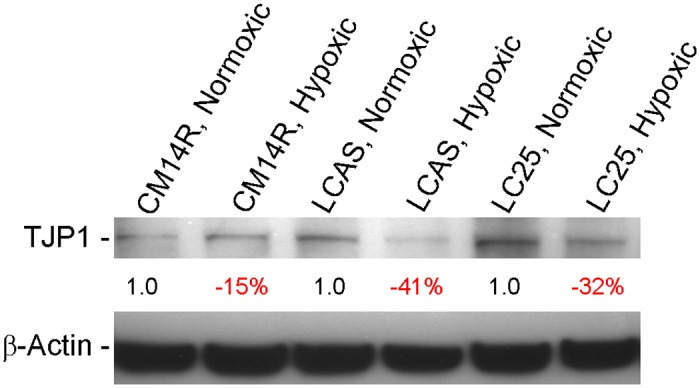
Western blot. TJP1 protein quantitative difference between the RG cell lines grown for 72 hours in normoxic versus hypoxic conditions. The quantitative differences of TJP1 protein calculated after the correction for actual protein loaded per lane using the β-Actin protein control.

The ability of “non-cancer stem cells” to generate “cancer stem cells” has recently been discussed [17]. Hypothetically, signaling events at the site where the onset of transformation occurs may trigger an epithelial-mesenchymal transition (EMT)-like process that would ultimately direct the RG cells toward transformation into tumor cells with neuro-ectodermal features [18–24]. Remarkably, N-cadherin, whose functional gain is considered a hallmark of EMT [21, 23, 24] is expressed in most of the tumor cells but not in the correspondent RG cells. (Fig 8J–8L). Using antibodies against TJP1and TJP3 we documented loss of tight junctions in the tumor cells, yet another hallmark of EMT [18, 24] (Fig 8A–8F). The same trend was documented for β-catenin, relocation of which from cell membranes to nucleus is also considered a hallmark of EMT [23, 24] (Fig 8G–8I). It is noteworthy that for β-catenin, as well as for TJP1and TJP3, such loss of expression was consistently observed around necrotic areas within tumors, opposite to the N-cadherin expression (Fig 8C, 8F, 8I and 8L). Since necrotic areas represent a hypoxic microenvironment, one might speculate that hypoxia could be one of the microenvironmental factors responsible for triggering of an (EMT)-like process, which was reported in a number of recent studies [20, 25, 26]. To test this hypothesis we cultured the RG cell lines for 72 hours in hypoxic versus normoxic conditions and subjected those to Western blot analyses of TJP1, TJP3 and N- Cadherin proteins. Apparently, the quantity of these proteins is very low in RG cell lines—considering the level of sensitivity of Western blot analysis. As a result, we were only able to detect the TJP1 protein. It is noteworthy, however, that the TJP1 protein was significantly down-regulated (15–41%) under hypoxic conditions (Fig 9).

**Fig 10:**
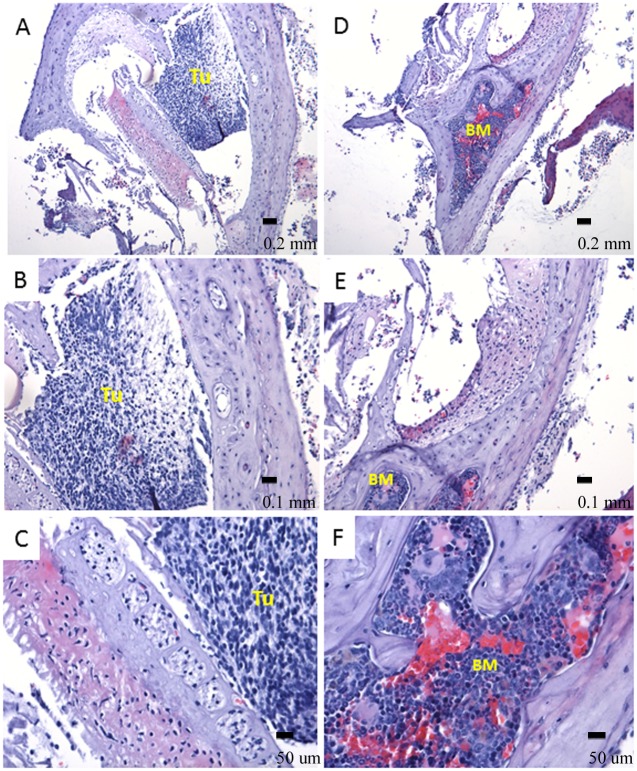
Ossification within tumor mass (LCAS-R—12 weeks post-injection). A, B, C. Mature and newly formed bone is observed in intimate correlation with the tumor mass (HE- 10X, 20X and 40X respectively). D, E and F. Bone marrow is observed within mature bone. It shows hematopoietic cells including erythrocytes, leucocytes and megakaryocytes in different stages of maturation suggesting active hematopoietic activity (HE- 10X, 20X and 40X respectively). BM- bone marrow, TU-tumor.

Interestingly, one of the tumors exhibited mature bone formation with bone marrow development, intermingling with tumor tissue, which was adhered to the base of the mouse brain (Fig 10). Similar findings were also reported in a number of clinical studies, particularly involving aggressive ependymoma and medulloblastoma [27, 28]. Such observation might be an additional indication of the occurrence of an EMT-like process. Future studies will determine whether EMT is indeed required for the observed transformation of radial glial cells in this model.

The results presented in this study clearly indicate that the alterations occurring in the transplanted cells that led to tumor formation are not inherited, but rather an acquired characteristic. Future work will focus on the identification of the molecular drivers and the microenvironmental factors that are responsible for committing otherwise normal RG cells to a malignant phenotype.

## Supporting Information

S1 File 3D imaging movie of the mouse brain using a confocal microscope at one month post—injection (LCAS-R). (AVI)
